# The prognostic impact of the tumour stroma fraction: A machine learning-based analysis in 16 human solid tumour types

**DOI:** 10.1016/j.ebiom.2021.103269

**Published:** 2021-03-09

**Authors:** Patrick Micke, Carina Strell, Johanna Mattsson, Alfonso Martín-Bernabé, Hans Brunnström, Jutta Huvila, Malin Sund, Fredrik Wärnberg, Fredrik Ponten, Bengt Glimelius, Ina Hrynchyk, Siarhei Mauchanski, Salome Khelashvili, Gemma Garcia-Vicién, David G. Molleví, Per-Henrik Edqvist, Aine O´Reilly, Sara Corvigno, Hanna Dahlstrand, Johan Botling, Ulrika Segersten, Agnieszka Krzyzanowska, Anders Bjartell, Jacob Elebro, Margareta Heby, Sebastian Lundgren, Charlotta Hedner, David Borg, Jenny Brändstedt, Hanna Sartor, Per-Uno Malmström, Martin Johansson, Björn Nodin, Max Backman, Cecilia Lindskog, Karin Jirström, Artur Mezheyeuski

**Affiliations:** a Department of Immunology, Genetics and Pathology, Uppsala University, Uppsala 751 85, Sweden; b Department of Oncology-Pathology, Karolinska Institutet, Stockholm, Sweden; c Department of Clinical Sciences Lund, Division of Pathology, Lund University, Lund, Sweden; d Department of Genetics and Pathology, Division of Laboratory Medicine, Region Skåne, Lund, Sweden; e Department of Pathology, University of British Columbia, Vancouver, Canada; f Department of Pathology, University of Turku, Turku, Finland; g Department of Surgical and perioperative sciences/Surgery, Umeå University, Umeå, Sweden; h Department of Surgery at Institute of Clinical Sciences, Sahlgrenska University Hospital Göteborg, Göteborg, Sweden; i City Clinical Pathologoanatomic Bureau, Minsk, Belarus; j N.N. Alexandrov National Cancer Centre of Belarus, 223040 Minsk, Belarus; k ProCURE, Program Against Cancer therapeutic Resistance, Catalan Institute of Oncology, Molecular Mechanisms and Experimental Therapy in Oncology Program (ONCOBELL), IDIBELL, L'Hospitalet de Llobregat, Barcelona, Catalonia, Spain; l Department of Surgical Sciences, Uppsala University, Uppsala 751 85, Sweden; m Department of Translational Medicine, Division of Urological Cancers, Lund University, Lund, Sweden; n Department of Clinical Sciences Lund, Division of Oncology and Therapeutic Pathology, Lund University, SE-221 00 Lund, Sweden; o Diagnostic Radiology, Department of Translational Medicine, Lund University, Skåne University Hospital, Lund, Sweden; p Department of Laboratory Medicine at Institute of Biomedicine, Sahlgrenska Universitety Hospital Göteborg, Göteborg, Sweden

## Abstract

**Background:**

The development of a reactive tumour stroma is a hallmark of tumour progression and pronounced tumour stroma is generally considered to be associated with clinical aggressiveness. The variability between tumour types regarding stroma fraction, and its prognosis associations, have not been systematically analysed.

**Methods:**

Using an objective machine-learning method we quantified the tumour stroma in 16 solid cancer types from 2732 patients, representing retrospective tissue collections of surgically resected primary tumours. Image analysis performed tissue segmentation into stromal and epithelial compartment based on pan-cytokeratin staining and autofluorescence patterns.

**Findings:**

The stroma fraction was highly variable within and across the tumour types, with kidney cancer showing the lowest and pancreato-biliary type periampullary cancer showing the highest stroma proportion (median 19% and 73% respectively). Adjusted Cox regression models revealed both positive (pancreato-biliary type periampullary cancer and oestrogen negative breast cancer, HR(95%CI)=0.56(0.34-0.92) and HR(95%CI)=0.41(0.17-0.98) respectively) and negative (intestinal type periampullary cancer, HR(95%CI)=3.59(1.49-8.62)) associations of the tumour stroma fraction with survival.

**Interpretation:**

Our study provides an objective quantification of the tumour stroma fraction across major types of solid cancer. Findings strongly argue against the commonly promoted view of a general associations between high stroma abundance and poor prognosis. The results also suggest that full exploitation of the prognostic potential of tumour stroma requires analyses that go beyond determination of stroma abundance.

**Funding:**

The Swedish Cancer Society, The Lions Cancer Foundation Uppsala, The Swedish Government Grant for Clinical Research, The Mrs Berta Kamprad Foundation, Sweden, Sellanders foundation, P.O.Zetterling Foundation, and The Sjöberg Foundation, Sweden.

## 1 Introduction

The malignant transformation of cancer cells is accompanied or even preceded by changes in the surrounding stroma [1]. The tumour stroma is histologically an integral part of cancer tissue and represents a complex composition of different cell types, including fibroblasts, immune cells and cells of the vasculature, and extracellular matrix [2]. There is strong evidence that tumorigenesis is controlled by the complex interaction between cancer cells and stroma elements and numerous experimental studies suggest that the tumour stroma exerts tumour-promoting effects [3], [4], [5], [6], [7], [8]. In line with this, many stroma features were identified that were related to tumour aggressiveness and ultimately impact patient outcome [3, [9], [10], [11]]. Consequently, the concept evolved that the development of the tumour stroma supports tumorigenesis and that these changes can be harnessed as prognostic or predictive biomarkers. Indeed, simply the amount of stroma in relation to the amount of tumour, can provide prognostic information. A higher amount of stroma, measured as stroma fraction assessed visually on diagnostic haematoxylin-eosin sections, was associated with shorter survival in many solid tumours (reviewed in [12]). However, the previous studies evaluated only one tumour type by the time and methodological aspects as well as publication bias complicate the comparison between different findings and makes a comprehensive interpretation difficult. The development of new image analysis systems [13, 14] provides the opportunity to evaluate the tumour stroma in a hitherto unrevealed resolution and accuracy. The aim of the study was to apply these advanced techniques to evaluate the tumour stroma fraction in the most common human solid tumour types.

**Research in context**

**Evidence before this study**

Solid malignant **tumours** are masses of malignant cells imbedded in a microenvironment of extracellular matrix, mesenchymal cells, endothelial cells, and infiltrating immune cells. These host elements of the tumour microenvironment are commonly called the tumour stroma and can support or suppress growth and dissemination of malignant cells. Multiple studies focused on specific elements of the tumour stroma. Some of these elements were largely reported as anti-tumorigenic (for example, lymphocytes) while others, like fibroblasts, were often considered pro-tumorigenic. Some studies, however, intended to analyse stroma as one entity, without sub-division into smaller elements. Such simplified approach could be of high value for clinical implementation. Indeed, a higher amount of stroma, measured as stroma fraction assessed visually by pathologist, predicted shorter survival in some solid tumours. However, previous studies are very heterogeneous with regard to methods used, and usually are limited to one tumour type. Additionally, a potential publication bias may be leading to the situation when negative results (no association with prognosis) are never published.

**Added value of this study**

Here we **developed** and applied a standardized technique to evaluate the tumour stroma fraction in surgically resected tumours. This large analysis includes 2732 individual cases which represent most common human solid tumour types.

**Implications of all the available evidence**

Our study **provides** a quantification of the tumour stroma fraction across the major types of solid cancer in human. Our findings challenge the common paradigm and suggest that high stroma fraction can be not only pro-tumorigenic but also anti-tumorigenic, depending on particular tumour type.

Alt-text: Unlabelled box

## 2 Methods

### 2.1 Material

Cancer cohorts were procured from different Scandinavian cancer centres (Table 1) and were provided as tissue micro arrays (TMA). The TMAs were constructed from resection specimens of operated cancer patients. Usually, each cancer tissue was represented by 1 to 2 tissue cores with core diameter between 1 and 1,5 mm. The individual cohorts used for the study were principally based on the location of the cancer, e.g. lung, breast or periampullary cancer, and have been published previously as described below. We further separated histologically, biologically and clinically different subgroups for our analysis, resulting in 16 different cancer groups. Detailed cohort description is available in Supplementary Materials.

**Table 1:** Tumour types used in the study.

	TUMOUR code	Tumour type	n	Ref.
**1**	RCC	Renal Cell Cancer	219	[35, 36]
**2**	CC	Colon Cancer	351	[37]
**3**	OVC	Ovarian Carcinoma	148	[38, 39]
**4**	ENC	Endometrial Cancer	301	[40, 41]
**5**	UBC	Urine Bladder Cancer	210	[42]
**6**	LUSC	Lung Squamous Cell Cancer	90	[43]
**7**	RC	Rectal Cancer	146	[37]
**8**	HGSC	High Grade Serous Ovarian Cancer	49	[44]
**9**	PACi	Periampullary Cancer, Intestinal type	61	[45, 46]
**10**	LUAD	Lung Adenocarcinoma	171	[43]
**11**	SC	Stomach Cancer	49	[47, 48]
**12**	GECA	Gastroesophageal Junction Adenocarcinoma	80	[47, 48]
**13**	BRC ER-	Breast Cancer, ER-negative	50	
**14**	PC	Prostate Cancer	241	[49]
**15**	BRC ER+	Breast Cancer, ER-positive	471	
**16**	PACpb	Periampullary Cancer, Pancreatobiliary type	95	[45, 46]
		Total meta-cohort	2732	

For the current study, only cases with invasive carcinomas and with available survival data were included in the analysis. Specific exclusion criteria may have been applied according to the guidance from the cohorts’ providers and are described in the Supplementary Materials.

### 2.2 Immuno-staining and image analysis

For the immunofluorescence staining, 4 µm thick sections were de-paraffinized, rehydrated and rinsed in distilled H_2_O. The staining procedure was described in details before [13, 15]. In short, a cocktail of antibodies was used to detect epithelial (cancer) tissue: anti‐E‐cadherin (Mouse/Clone 36, BD Biosciences/610182, 1:5000), anti‐pan Cytokeratin (Mouse/[C‐11], Abcam, San Francisco/ab7753, 1:1000) and anti‐pan Cytokeratin (Mouse/AE1/AE3, Thermo Fisher Scientific/MA5‐13156, 1:500). The staining was visualized by the fluorophore Opal 690 (FP1497001KT, Akoya Biosciences, MA, USA). Stained tissue was scanned with the Vectra Polaris system (Akoya Biosciences) at resolution of 0.5 μm per 1 pixel. The multiplex staining included also staining with other markers for different subsets of immune cells (Mezheyeuski in preparation) but were not used in this study.

The images, each representing a TMA core, were processed with the inForm Analysis software (Akoya Biosciences), where tissue was classified into three types: Epithelial (Tumor), Stroma and blank areas. Machine learning function based on the built-in inForm software (Akoya Biosciences) was applied for tissue segmentation using expression data of cytokeratin/E-cadherin, DAPI staining and spectrally unmixed autofluorescence. Each of 7918 images was reviewed by pathologists (PM, IH, SM, SK, AM) and by HistoOne AB (Uppsala, Sweden): samples which did not contain invasive tumour or were damaged, were excluded completely while other samples were curated manually for the exclusion of necrosis or artefacts. Additionally, each sample was quality controlled with regard to the presence of malignant tissue (with associated stroma) of invasive cancer and regions of cancer *in situ* or regions with non-malignant tissue and associated stroma were manually excluded.

The images were coded according to the position of the TMA core on the slide. The ‘key’ to match TMAs to patient IDs was received from cohort providers after the curation was completed. Thus, all involved pathologists were blinded concerning any clinical data, except the affiliation of the sample to certain cohort. The TMA/image to patient matching procedure was performed at the final stages of the data processing.

### 2.3 Data processing

Data processing was performed using R software (version 3.5.1). The stroma fraction was computed: stroma fraction = stroma area / (stroma area + epithelial area). In cases where more than one TMA core represented a single tumour, the calculation of the stroma fraction was processed by assuming the existing material as one sample (i.e. to avoid unequal impact from cores of different size).

### 2.4 Statistics

Statistical analyses were performed using R (version 3.5.1) and SPSS V20 (SPSS Inc., Chicago, IL). Overall survival, defined as time from surgery for primary tumour to date of death from any cause, was the defined analytical endpoint. Patients were censored in case of discontinued follow-up. Kaplan Meier survival estimates were computed and visualized using the “*survival”* and “*survminer”* packages for R. Log-Rank test was used for comparisons between patient groups. For the survival analyses for each tumour type, patients were grouped based on median stroma fraction as cut-off. Additionally, to control for potential bias with improper cut-off selection, stroma fraction values were used as continuous values, rounded to first decimal and tested in Cox regression models. To estimate relative hazards in both univariable and multivariable models, a Cox proportional hazards model was used with dichotomized stroma fraction (R *“survival”* package). P values < 0.05 were considered as statistically significant.

The proportional hazards assumption was assessed graphically and tested with Schoenfeldt's test using cox.zph function (R *“survival”* package). The proportional hazards assumption was violated in intestinal type periampullary cancer for stroma fraction in univariable model and for covariables stroma fraction and pT stage in the multi-variable model. The scaled Schoenfeld residuals against the transformed time were visualised by function ggcoxzph() (R *“survminer”* package) and a knee point at time-scale 70 was observed. We therefore created modified models with (co)variables stroma fraction and pT stage split by time segments (0 to 70, and 70 to max).

*Clinicopathological parameters inclusion in multivariable models.* Patient age at diagnosis was included in all multivariable models as co-variable, and was dichotomized with the median as cut-off. The exception was made for breast cancer (ER+ and ER-) due to known non-linear impact of the patient age on survival: the age groups for the multivariable analysis in breast cancer were set up as <=50years, >50-<65 and >=65 years. WHO performance status, pT stage, differentiation grade were used as categorical variables. pN and pM stage were dichotomized into N0 vs N>=1 or M0 vs M>=1 respectively. Resection margin status with regard to the presence of residual tumour was accessed as categorical variable of three categories: R0 (no residual tumour), R1 (microscopic residual tumour) or R2 (macroscopic residual tumour). Adjuvant and pre-surgical therapy were assessed as present or absent, without separation for specific treatment type. As a general rule, missing values of each variable were treated as separate category. Exceptions were done for some cases (colon cancer, prostate cancer and breast cancer) which had missing variables for more than one clinicopathological parameter - these cases were excluded from multivariable models.

### 2.5 Role of the funding source

The funders did not have any role in the design of the study, data collection, data analysis or data interpretation, or writing of the manuscript. The corresponding author had full access to all the data in the study and had final responsibility for the decision to submit for publication.

## 3 Results

The aim of our study was to provide a comprehensive and objective analysis of the tumour stroma amount in 16 different solid cancer types, including in total 2732 cancer patients (Table 1, Suppl. Material, Suppl. Table 1). Using an immunofluorescent staining for epithelial markers and a machine learning based image analysis we calculated the stroma fraction for each individual cancer cases (Suppl. Fig. 1).

**Fig. 1:**
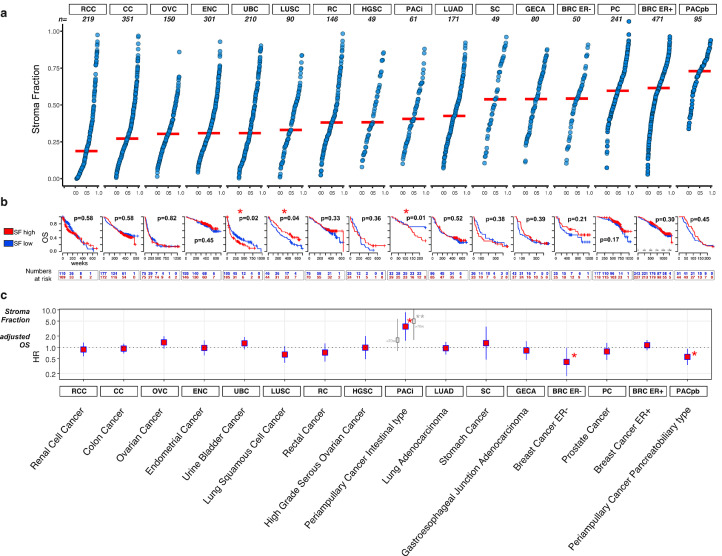
Stroma fraction in solid cancer types. (a) The empirical cumulative distribution plots of the stroma fractions in 16 cancer entities. Each dot represents an individual sample, red horizontal lines indicate the median value of stroma fraction in the respective cancer type. (b) Kaplan–Meier plots illustrate the overall survival in each cohort. Stroma fraction (SF) dichotomized into stroma rich (red) and stroma poor (blue) groups (cut-off = median stroma fraction). The p-values refer to the log-rank test. (c) Forest plots of multivariable Cox regression models representing hazard ratios of the association between stroma fraction and overall survival. For each tumour type, a square indicates the hazard ratio and the line on either side of the square demonstrates 95% confidence interval. A higher hazard ratio indicates that a higher stroma fraction is associated with a shorter survival. Co-variables were selected individually for each tumour type, depending on their availability and known clinical importance (Detailed information is shown in Suppl. Fig). The tumour cohorts are described in detail in the Supplementary Material. **The proportional hazards assumption was violated in PACi and therefore the illustrated hazard ratio should be interpreted as the mean value over time; corrected model, split into time segments before and after 70 weeks, demonstrated by grey colour.

When comparing different tumour types, we observed major differences in the median levels of stroma fraction, ranging from less than 25% in renal cell carcinoma to over 70% in pancreatobiliary type periampullary cancer (Fig. 1a). Likewise, within individual tumour types the stroma fraction varied considerably, displaying both stroma-poor and stroma-rich cases.

To analyse the impact of the amount of stroma on overall survival, the stroma fraction value was dichotomized into stroma high and low groups, using the median as cut-off in each tumour type. Kaplan-Meier analysis revealed that a higher stroma fraction was associated with shorter survival in urinary bladder cancer and in intestinal type periampullary cancer (Fig. 1b, Suppl. Table 2). On the other hand, in lung squamous cell cancer a higher stroma fraction was associated with improved survival. These associations were also observed in a univariable Cox regression models using continuous stroma fraction values. After statistical correction for intestinal type periampullary cancer the association was only demonstrated after the time segment of 70 weeks (Suppl. Table 3 and Material and Methods).

Subsequently, we performed survival analyses adjusted for established prognostic factors, which were selected for each tumour type depending on relevance and data availability (Suppl. Fig. 2). In this multivariable Cox regression model, the adverse prognostic impact of higher stroma fraction remained significant only in intestinal type periampullary cancer, HR(95%CI)=3.59(1.49-8.62), when illustrated as the mean value over time (Fig. 1c). Interesting, similar to univariable analysis, when the model was split to time segments, stroma fraction demonstrated prognostic impact only after 70 weeks of patients survival after surgery (HR(95%CI)=5.78(1.87-18.40), p=0.0024), but not during first 70 weeks (HR(95%CI)=1.57(0.43-5.75), p=0.0024) (Fig. 1c, demonstrated by grey colour). Contrarily, in periampullary cancer pancreatobiliary type, a higher stroma fraction was associated with improved survival, HR(95%CI)=0.56(0.34-0.92). The same positive association was observed for oestrogen negative breast cancer, HR(95%CI)=0.41(0.17-0.98) (Fig. 1c).

## 4 Discussion

The study provides a comprehensive, objective assessment of the tumour fraction in the main solid cancer types. Our data demonstrate that the fraction of tumour stroma is highly variable between and within solid cancer types. In most cancer types (13 of 16) no significant associations of the tumour stroma fraction and survival were observed in the multivariable analysis. Only in one cancer type the stroma fraction indicated prognostic unfavourable impact (periampullary cancer intestinal type) and in two cancer types favourable impact (periampullary cancer pancreatobiliary type and oestrogen receptor negative breast cancer).

The majority of previous studies have indicated a negative prognostic impact of a dense tumour stroma, most often measured as the tumour stroma ratio (TSR), e.g. in colorectal cancer [16], [17], [18], gastric cancer [19], breast cancer [20, 21], cervical cancer [22], and oesophageal squamous cell cancer [23]. This association was also confirmed in a meta-analysis including 14 studies of different cancer types [12]. The studies are mainly based on careful visual annotations of whole tumour sections and included a selection of the most desmoplastic regions in haematoxylin and eosin stained tissue: a method developed by the group of Mesker [24].

Our approach was different as the tissue samples were not preselected to present stroma rich areas, but rather consisted of regular representative tumour regions. Moreover, our stroma evaluation was based on highly specific immunofluorescence staining supplemented by an objective image analysis, and was standardized for all analysed cohorts. This allowed a reliable comparison between the different tumour types.

With this approach, the previously described unfavourable prognostic impact of the tumour stroma fraction was only observed in two cancer types in the univariable and only one in the adjusted analysis. However, for three cancer types, a high stroma fraction was found to correlate to an improved prognosis. Accordingly, isolated previous studies in different cancer types indicated that higher stroma content was associated with longer survival [25], [26], [27], [28]. These and our studies clearly challenge the previous paradigm of the “bad tumour stroma” and support more recent experimental findings that reveal a tumour suppressive role of cancer-associated fibroblasts in pancreatic cancer models [29], [30], [31], indicating that the stroma can also act as a barrier against tumour progression, invasion and development of distant metastasis. The specific evaluation of stroma signalling that inhibit tumour development can provide novel strategies that supplement current armamentarium of cancer therapy.

It should be noted that our strategy has certain limitations. TMA samples represent only a minor fraction of the whole tumour tissue and may therefore not be able to reflect adequately all aspects of tumour architecture. Additionally, in this study we did not apply the specific methodology for the description of the stroma abundance, developed by Mesker *et al*. [24], i.e. we did not screen through the tissue sample for the regions of most extensive fibrosis. Therefore, our results should be interpreted with caution and clearly delineated from the guided approach.

In conclusion, crude measurement of the tumour stroma content might be insufficient for the purpose of prognostication, and it is likely that other stromal qualities, including mechanical features, extracellular matrix components or immune cell composition have overriding importance in the tumorigenic process [6, [32], [33], [34]]. Nevertheless, we believe that our comprehensive analysis contributes, on an elementary level, with novel perspectives on the tumour-stroma interaction and serves as a strong starting point for further focused studies aimed at elucidating the role of the tumour stroma fraction in cancer.

## 5 Data sharing

The data regarding the methodology, image analysis, curation and data processing as well as raw-data of stroma fraction is available from the corresponding author by request. The data related to cohort characteristics (clinical and pathological data etc.) can be requested from cohort providers: the requests can be sent vie corresponding author or directly to cohort providers (see reference publications in Table 1).

## CRediT authorship contribution statement

**Patrick Micke:** Data curtion, Funding acquisition, Resources, Supervision, Writing – original draft, Writing – review & editing. **Carina Strell:** Investigation, Writing – original draft, Writing – review & editing. **Johanna Mattsson:** Data curtion. **Alfonso Martín-Bernabé:** Data curtion, Writing – review & editing. **Hans Brunnström:** Resources, Writing – review & editing. **Jutta Huvila:** Resources, Writing – review & editing. **Malin Sund:** Resources, Writing – review & editing. **Fredrik Wärnberg:** Resources, Writing – review & editing. **Fredrik Ponten:** Resources, Writing – review & editing. **Bengt Glimelius:** Resources, Writing – review & editing. **Ina Hrynchyk:** Data curtion, Writing – review & editing. **Siarhei Mauchanski:** Data curtion. **Salome Khelashvili:** Data curtion. **Gemma Garcia-Vicién:** Data curtion, Writing – review & editing. **David G. Molleví:** Formal analysis, Writing – review & editing. **Per-Henrik Edqvist:** Resources, Writing – review & editing. **Aine O´Reilly:** Data curtion, Writing – review & editing. **Sara Corvigno:** Resources, Writing – review & editing. **Hanna Dahlstrand:** Resources, Writing – review & editing. **Johan Botling:** Resources, Writing – review & editing. **Ulrika Segersten:** Resources, Writing – review & editing. **Agnieszka Krzyzanowska:** Resources, Writing – review & editing. **Anders Bjartell:** Resources, Writing – review & editing. **Jacob Elebro:** Resources, Writing – review & editing. **Margareta Heby:** Resources, Writing – review & editing. **Sebastian Lundgren:** Investigation, Resources, Writing – review & editing. **Charlotta Hedner:** Investigation, Writing – review & editing. **David Borg:** Investigation. **Jenny Brändstedt:** Investigation. **Hanna Sartor:** Resources, Writing – review & editing. **Per-Uno Malmström:** Formal analysis, Resources, Writing – review & editing. **Martin Johansson:** Resources, Writing – review & editing. **Björn Nodin:** Resources, Writing – review & editing. **Max Backman:** Data curtion, Investigation, Writing – review & editing. **Cecilia Lindskog:** Resources, Writing – review & editing. **Karin Jirström:** Conceptualization, Methodology, Resources, Writing – review & editing. **Artur Mezheyeuski:** Conceptualization, Data curtion, Formal analysis, Investigation, Methodology, Project administration, Resources, Software, Supervision, Validation, Visualization, Writing – original draft, Writing – review & editing.

## Declaration of Competing Interest

Conflict of interest statement: Artur Mezheyeuski, Carina Strell and Patrick Micke own shares in the company HistoOne AB, Uppsala, Sweden, which infrastructure was used for the pathology assessment of tissue samples in the study. The other authors have no conflicts of interest to disclose.

## Appendix Supplementary materials

Image, application 1
