# Supplementary material for: The prognostic impact of the tumour stroma fraction: A machine learning-based analysis in 16 human solid tumour types
Source: eBioMedicine. 2021 Mar 9;65:103269. doi: 10.1016/j.ebiom.2021.103269 (PMC7960932; doi:10.1016/j.ebiom.2021.103269)
Supplement: Supplementary file 1 [file mmc1.docx]

**Patient Cohorts**

*Colon Cancer (CC) and Rectal Cancer (RC). -* The colorectal cancer TMAs were derived from a population-based CRC collection which was obtained in the context of the U-CAN initiative (<http://www.u-can.uu.se/about-u-can/>) (1) (Ethical approval Uppsala, 116/07; 419/15).

*Periampullary Cancer of intestinal (PACi) and pancreatobiliary (PACpb) type. -* The TMA cohort consisted of primary tumour samples collected retrospectively from patients who underwent pancreaticoduodenectomy for periampullary adenocarcinoma in the University hospitals in Lund and Malmö, Sweden between 2001 and 2011 (2, 3) (Ethical approval Lund, 445/07). Neoadjuvant therapy was considered as exclusion criteria and prospective cases were not included in the current study.

*Ovarian Cancer (OVC). -* The cohort consisted of the TMAs, comprised from invasive ovarian cancer cases of two prospective, population-based cohorts: the Malmö Diet and Cancer Study and Malmö Preventive Project (4-6), (Ethical approval Lund, 445/07).

*High Grade Serous Ovarian Cancer (HGSC). -* A HGSC TMA was built from a chemo-naive cohort of patients diagnosed with HGSC between 2002 and 2006, which were treated at Karolinska University Hospital, Stockholm, Sweden (7), (Ethical approval Stockholm, 2016/551–32).

*Renal Clear Cell (RCC). -* The TMA cohort consists of primary renal cell cancer samples from patients diagnosed between 1978 and 1996 in Skåne University Hospital Malmö (8, 9), (Ethical approval Malmo, 282/07).

*Urine Bladder Cancer (UBC). -* The TMA cohort consists of 357 prospectively collected primary urothelial tumours from patients operated at the Uppsala University Hospital between 1984 and 2005 (10), (Ethical approval Uppsala, 2005/143).

*Endometrial Cancer (ENC). -* The TMA cohort consists of primary uterine carcinomas from patients undergoing treatment at Turku University Hospital, Finland between 2004 and 2007 (11, 12), (Ethical approval Helsinki, 2016/010).

*Non Small Cell Lung Cancer presented as Lung Squamous Cell Carcinoma (LUSC) and Lung Adenocarcinoma (LUAD). -* This cohort included 357 patients with Non-Small Cell Lung Cancer who underwent surgery at Uppsala University Hospital, Sweden between 2006 and 2010 (13), (Ethical approval Uppsala, 532/12).

*Gastroesophageal Junction Adenocarcinoma (GECA) and Stomach Cancer (SC). -* The TMA cohort included 174 patients with chemoradiotherapy-naïve oesophageal and gastric tumours (including the esophagogastric junction) who underwent surgical resection at the University Hospitals of Lund and Malmö between 2006 and 2010 (14, 15). The location of the primary tumour was defined by endoscopy records. For the current study the cohort was split into Gastroesophageal Junction Adenocarcinoma (tumours with the location in a lower third of the oesophagus, as well as in cardia Siewert type 1 and 2) and Stomach Cancer (location cardia Siewert type 3, stomach corpus and antrum), (Ethical approval Lund, 445/07).

*Prostate Cancer (PC). -* This population‐based TMA cohort included 341 hormone-naïve prostate cancer who underwent open radical prostatectomy between 1998 and 2006 at the Department of Urology, Skåne University Hospital, Malmö, Sweden (16), (Ethical approval Lund, 494/05).

*Breast Cancer (BRC ER- and ER+). -* The tissue collection is a population-based cohort from Uppsala and Västerås health care regions including women with an invasive breast cancer, 15 mm or less, with or without an *in situ* component, diagnosed between 1987 and 2004 (n=798). Also, larger invasive tumours with an *in situ* component noted in the histopathology report were included (n=139). Each TMA core was inspected visually and cases which only contained *in situ* component with no (or minor) invasive tumour were excluded from current study. Tissue samples which included less than 50% of *in situ* component were included, but the tissue regions containing *in situ* lesion were manually excluded from further analysis by pathologists, (Ethical approval Uppsala, Umeå, 2005/118/2).

**Supplementary Figure 1:** Image analysis pipeline and representative illustrations of compartment segmentation in different tumour cohorts

**Supplementary Figure 2:** Grey squares indicate the co-variables that were included in the multivariate Cox regression model for each tumour type (see Fig. 1c).

**Supplementary Table 1: Clinicopathological characteristics of the tumour cohorts used in the study**

|  | Renal Cell Cancer | Colon  Cancer | Ovarian Cancer | Endometrial Cancer | Urine Bladder Cancer | Lung Squamous Cell Cancer | Rectal  Cancer | High Grade Serous Ovarian Cancer | Periampullary Cancer Intestinal type | Lung Adenocarcinoma | Stomach Cancer | Gastroesophageal Junction Adenocarcinoma | Breast  Cancer  ER- | Prostate Cancer | Breast  Cancer  ER+ | Periampullary Cancer Pancreatobiliary type |
| --- | --- | --- | --- | --- | --- | --- | --- | --- | --- | --- | --- | --- | --- | --- | --- | --- |
| Tumour code | RCC | CC | OVC | ENC | UBC | LUSC | RC | HGSC | PACi | LUAD | SC | GECA | BRC ER- | PC | BRC ER+ | PACpb |
| **Patient sample size and median survival time** |  |  |  |  |  |  |  |  |  |  |  |  |  |  |  |  |
| Total number of patients (%) | 219 (8%) | 351 (12,8%) | 150 (5,5%) | 301 (11%) | 210 (7,7%) | 90 (3,3%) | 146 (5,3%) | 49 (1,8%) | 61 (2,2%) | 171 (6,3%) | 49 (1,8%) | 80 (2,9%) | 50 (1,8%) | 241 (8,8%) | 471 (17,2%) | 95 (3,5%) |
| Median survival time^a^ ± SD (weeks) | 251 ± 25,24 | 367 ± 38,98 | 151 ± 22,20 | 543,17 ± 14,03^b^ | 142 ± 25,38 | 170,63 ± 64,98 | 494 ± 54,01 | 190 ± 35,57 | 176,25 ± 10,14^b^ | 252 ± 37,85 | 93 ± 30,09 | 123 ± 22,92 | 792 ± 207,29 | 958 ± 100,28 | 1064 ± 34,01 | 100 ± 6,09 |
| **Age at diagnosis** |  |  |  |  |  |  |  |  |  |  |  |  |  |  |  |  |
| mean ± SD | 65,29 ± 11,46 | 70,14 ± 12,40 | 63,43 ± 8,29 | 65,99 ± 10,44 | 72,68 ± 11,23 | 68,32 ± 7,45 | 69,74 ± 10,95 | 64,06 ± 10,20 | 64,05 ± 9,55 | 66,73 ± 7,36 | 74,35 ± 10,99 | 68,51 ± 10,82 | - | 63,22 ± 5,74 | - | 66,49 ± 7,57 |
| median ± SD | 67 ± 11,46 | 70 ± 12,40 | 62 ± 8,29 | 66 ± 10,44 | 73 ± 11,23 | 68,50 ± 7,45 | 69 ± 10,95 | 63 ± 10,20 | 66 ± 9,55 | 66 ± 7,36 | 78 ± 10,99 | 66 ± 10,82 | - | 63 ± 5,74 | - | 67 ± 7,57 |
| ≤ median | 111 (50,7%) | 178 (50,7%) | 76 (50,7%) | 155 (51,5%) | 107 (51%) | 45 (50%) | 76 (52,1%) | 25 (51%) | 31 (50,8%) | 87 (50,9%) | 25 (51%) | 41 (51,3%) | - | 123 (51%) | - | 48 (50,5%) |
| > median | 108 (49,3%) | 173 (49,3%) | 74 (49,3%) | 146 (48,5%) | 103 (49%) | 45 (50%) | 70 (47,9%) | 24 (49%) | 30 (49,2%) | 84 (49%) | 24 (49%) | 39 (48,8%) | - | 118 (49%) | - | 47 (49,5%) |
| ≤ 50 | - | - | - | - | - | - | - | - | - | - | - | - | 15 (30%) | - | 122 (25,9%) | - |
| >50-<65 | - | - | - | - | - | - | - | - | - | - | - | - | 16 (32%) | - | 177 (37,6%) | - |
| ≥ 65 | - | - | - | - | - | - | - | - | - | - | - | - | 19 (38%) | - | 172 (36,5%) | - |
| **Sex** |  |  |  |  |  |  |  |  |  |  |  |  |  |  |  |  |
| Female | 100 (45,7%) | 165 (47%) | 150 (100%) | 301 (100%) | 53 (25,2%) | 38 (42,2%) | 59 (40,4%) | 49 (100%) | 34 (55,7%) | 96 (56,1%) | 16 (32,7%) | 13 (16,3%) | 50 (100%) | - | 471 (100%) | 43 (45,3%) |
| Male | 119 (54,3%) | 186 (53%) | - | - | 157 (74,8%) | 52 (57,8%) | 87 (59,6%) | - | 27 (44,3%) | 75 (43,9%) | 33 (67,3%) | 67 (83,8%) | - | 241 (100%) | - | 52 (54,7%) |
| **WHO performance status** |  |  |  |  |  |  |  |  |  |  |  |  |  |  |  |  |
| 0 | 0 | 0 | 0 | 0 | 0 | 50 (55,6%) | 0 | 22 (44,9%) | 0 | 109 (63,7%) | 0 | 0 | 0 | 0 | 0 | 0 |
| 1 | 0 | 0 | 0 | 0 | 0 | 40 (44,4%) | 0 | 22 (44,9%) | 0 | 59 (34,5%) | 0 | 0 | 0 | 0 | 0 | 0 |
| 2 | 0 | 0 | 0 | 0 | 0 | 0 | 0 | 4 (8,2%) | 0 | 3 (1,8%) | 0 | 0 | 0 | 0 | 0 | 0 |
| 3 | 0 | 0 | 0 | 0 | 0 | 0 | 0 | 1 (2%) | 0 | 0 | 0 | 0 | 0 | 0 | 0 | 0 |
| Missing data | 219 (100%) | 351 (100%) | 150 (100%) | 301 (100%) | 210 (100%) | 0 | 146 (100%) | 0 | 61 (100%) | 0 | 49 (100%) | 80 (100%) | 50 (100%) | 241 (100%) | 471 (100%) | 95 (100%) |
| **pT classification** |  |  |  |  |  |  |  |  |  |  |  |  |  |  |  |  |
| pT1 | 0 | 27 (7,7%) | 0 | 0 | 103 (49%) | 35 (38,9%) | 11 (7,5%) | 0 | 4 (6,6%) | 82 (48%) | 4 (8,2%) | 3 (3,8%) | 44 (88%) | 0 | 421 (89,4%) | 2 (2,1%) |
| pT2 | 0 | 17 (4,8%) | 0 | 0 | 87 (41,4%) | 40 (44,4%) | 43 (29,5%) | 0 | 10 (16,4%) | 66 (38,6%) | 10 (20,4%) | 13 (16,3%) | 6 (12%) | 122 (50,6) | 23 (4,9%) | 9 (9,5%) |
| pT3 | 0 | 218 (62,1%) | 0 | 0 | 15 (7,1%) | 12 (13,3%) | 75 (51,4%) | 0 | 25 (41%) | 18 (10,5%) | 24 (49%) | 54 (67,5%) | 0 | 111 (46,1) | 4 (0,8%) | 69 (72,6%) |
| pT4 | 0 | 88 (25,1%) | 0 | 0 | 5 (2,4%) | 3 (3,3%) | 10 (6,8%) | 0 | 22 (36,1%) | 5 (2,9%) | 11 (22,4%) | 10 (12,5%) | - | 2 (0,8%) | - | 15 (12,8%) |
| Missing data | 219 (100%) | 1 (0,3%) | 150 (100%) | 301 (100%) | 0 | 0 | 7 (4,8%) | 49 (100%) | 0 | 0 | 0 | 0 | 0 | 6 (2,5%) | 23 (4,9%) | 0 |
| **pN classification** |  |  |  |  |  |  |  |  |  |  |  |  |  |  |  |  |
| pN0 | 187 (85,4%) | 163 (46,4%) | 0 | 0 | 32 (15,2%) | 76 (84,4%) | 94 (64,4%) | 0 | 33 (54,1%) | 124 (72,5%) | 21 (42,9%) | 16 (20%) | - | 0 | - | 27 (28,4%) |
| pN1 | 24 (11%) | 187 (53,3%) | 0 | 0 | 10 (4,8%) | 11 (12,2%) | 43 (29,5%) | 0 | 28 (45,9%) | 20 (11,7%) | 12 (24,5%) | 15 (18,8%) | - | 0 | - | 68 (71,6%) |
| pN2 | 0 | 0 | 0 | 0 | 0 | 3 (3,3%) | 0 | 0 | 0 | 27 (15,8%) | 9 (18,4%) | 24 (30%) | - | 0 | - | 0 |
| pN3 | 0 | 0 | 0 | 0 | 0 | 0 | 0 | 0 | 0 | 0 | 7 (14,3%) | 25 (31,3%) | - | 0 | - | 0 |
| Missing data | 8 (3,7%) | 1 (0,3%) | 150 (100%) | 301 (100%) | 168 (80%) | 0 | 9 (6,2%) | 49 (100%) | 0 | 0 | 0 | 0 | 50 (100%) | 241 (100%) | 471 (100%) | 0 |
| **pN classification, reduced** |  |  |  |  |  |  |  |  |  |  |  |  |  |  |  |  |
| pN0 | 187 (85,4%) | 163 (46,4%) | 0 | 0 | 32 (15,2%) | 76 (84,4%) | 94 (64,4%) | 0 | 33 (54,1%) | 124 (72,5%) | 21 (42,9%) | 16 (20%) | - | 0 | - | 27 (28,4%) |
| ≥ pN1 | 24 (11%) | 187 (53,3%) | 0 | 0 | 10 (4,8%) | 14 (15,6%) | 43 (29,5%) | 0 | 28 (45,9%) | 47 (27,5%) | 28 (57,1%) | 64 (80%) | - | 0 | - | 68 (71,6%) |
| Missing data | 8 (3,7%) | 1 (0,3%) | 150 (100%) | 301 (100%) | 168 (80%) | 0 | 9 (6,2%) | 49 (100%) | 0 | 0 | 0 | 0 | 50 (100%) | 241 (100%) | 471 (100%) | 0 |
| **pM classification** |  |  |  |  |  |  |  |  |  |  |  |  |  |  |  |  |
| pM0 | 177 (80,8%) | 289 (82,3%) | 0 | 0 | 60 (28,6%) | 0 | 130 (89%) | 0 | 0 | 0 | 44 (89,8%) | 74 (92,5%) | 0 | 0 | 0 | 0 |
| pM1 | 39 (17,8%) | 60 (17,1%) | 0 | 0 | 33 (15,7%) | 0 | 13 (8,9%) | 0 | 0 | 0 | 2 (4,1%) | 1 (1,3%) | 0 | 0 | 0 | 0 |
| pM2 | 1 (0,5) | 0 | 0 | 0 |  | 0 | 0 | 0 | 0 | 0 | 3 (6,1%) | 5 (6,3%) | 0 | 0 | 0 | 0 |
| Missing data | 2 (0,9%) | 2 (0,6%) | 150 (100%) | 301 (100%) | 117 (55,7%) | 90 (100%) | 3 (2,1%) | 49 (100%) | 61 (100%) | 171 (100%) | 0 | 0 | 50 (100%) | 241 (100%) | 471 (100%) | 95 (100%) |
| **pM classification, reduced** |  |  |  |  |  |  |  |  |  |  |  |  |  |  |  |  |
| pM0 | 177 (80,8%) | 289 (82,3%) | 0 | 0 | 60 (28,6%) | 0 | 130 (89%) | 0 | 0 | 0 | 44 (89,8%) | 74 (92,5%) | 0 | 0 | 0 | 0 |
| ≥ pM1 | 40 (18,3%) | 60 (17,1%) | 0 | 0 | 33 (15,7%) | 0 | 13 (8,9%) | 0 | 0 | 0 | 5 (10,2%) | 6 (7,5%) | 0 | 0 | 0 | 0 |
| Missing data | 2 (0,9%) | 2 (0,6%) | 150 (100%) | 301 (100%) | 117 (55,7%) | 90 (100%) | 3 (2,1%) | 49 (100%) | 61 (100%) | 171 (100%) | 0 | 0 | 50 (100%) | 241 (100%) | 471 (100%) | 95 (100%) |
| **Clinical stage at diagnosis** |  |  |  |  |  |  |  |  |  |  |  |  |  |  |  |  |
| 1 | 0 | 36 (10,3%) | 26 (17,3%) | 244 (81,1%) | 0 | 58 (64,4%) | 47 (32,2%) | 0 | 0 | 104 (60,8%) | 0 | 0 | 0 | 0 | 0 | 0 |
| 2 | 0 | 116 (33%) | 18 (12%) | 9 (3%) | 0 | 26 (28,9%) | 46 (31,5%) | 1 (2%) | 0 | 28 (16,4%) | 0 | 0 | 0 | 0 | 0 | 0 |
| 3 | 0 | 138 (39,3%) | 73 (48,7%) | 40 (13,3%) | 0 | 6 (6,7%) | 38 (26%) | 36 (73,5%) | 0 | 32 (18,7%) | 0 | 0 | 0 | 0 | 0 | 0 |
| 4 | 0 | 60 (17,1%) | 20 (13,3%) | 8 (2,7%) | 0 | 0 | 13 (8,9%) | 12 (24,5%) | 0 | 7 (4,1%) | 0 | 0 | 0 | 0 | 0 | 0 |
| Missing data | 219 (100%) | 1 (0,3%) | 13 (8,7%) | 0 | 210 (100%) | 0 | 2 (1,4%) | 0 | 61 (100%) | 0 | 49 (100%) | 80 (100%) | 50 (100%) | 241 (100%) | 471 (100%) | 95 (100%) |
| **Differentiation Grade** |  |  |  |  |  |  |  |  |  |  |  |  |  |  |  |  |
| G1 Well differentiated (Low grade) | 96 (43,8%) | 241 (68,7%) | 7 (4,7%) | 250 (83,1%) | 5 (2,4%) | 0 | 113 (77,4%) | - | 31 (50,8%) | 0 | 32 (65,3%) | 46 (57,5%) | 4 (8%) | 103 (42,7%) | 198 (42%) | 59 (62,1%) |
| G2 Moderately differentiated (Intermediate grade) | 74 (33,8%) | - | 37 (24,7%) | - | - | 0 | - | - | - | 0 | - | - | 17 (34%) | 84 (34,9%) | 223 (47,3%) | - |
| G3-G4 Poorly differentiated (High grade) | 47 (21,4%) | 69 (19,7%) | 106 (70,7%) | 51 (16,9%) | 205 (97,6%) | 0 | 13 (8,9%) | 49 (100%) | 30 (49,2%) | 0 | 17 (34,7%) | 34 (42,5%) | 29 (58%) | 38 (15,7%) | 49(10,4%) | 36 (37,9%) |
| G5 Very poorly differentiated | - | - | - | - | - | - | - | - | - | - | - | - | - | 14 (5,8%) | - | - |
| Missing data | 2 (0,9%) | 41 (11,7%) | 0 | 0 | 0 | 90 (100%) | 20 (13,7%) | 0 | 0 | 171 (100%) | 0 | 0 | 0 | 2 (0,8%) | 1 (0,2%) | 0 |
| **Tumour size (Breast cancer only)** |  |  |  |  |  |  |  |  |  |  |  |  |  |  |  |  |
| >10 mm | - | - | - | - | - | - | - | - | - | - | - | - | 29 (58%) | - | 272 (57,7%) |  |
| ≤10 mm | - | - | - | - | - | - | - | - | - | - | - | - | 21 (42%) | - | 199 (42,3%) |  |
| **Resection margin** |  |  | 150 (100%) |  |  |  |  |  |  |  |  |  |  |  |  |  |
| R0 | 0 | 0 | 0 | 0 | 0 | 0 | 0 | 0 | 17 (27,9%) | 0 | 34 (69,4%) | 52 (65%) | 0 | 0 | 0 | 6 (6,3%) |
| R1 | 0 | 0 | 0 | 0 | 0 | 0 | 0 | 0 | 13 (21,3%) | 0 | 10 (20,4%) | 26 (32,5%) | 0 | 0 | 0 | 70 (73,7%) |
| R2 | 0 | 0 | 0 | 0 | 0 | 0 | 0 | 0 | 0 | 0 | 5 (10,2%) | 2 (2,5%) | 0 | 0 | 0 | 0 |
| Rx | 0 | 0 | 0 | 0 | 0 | 0 | 0 | 0 | 31 (50,8%) | 0 | 0 | 0 | 0 | 0 | 0 | 19 (20%) |
| Missing data | 219 (100%) | 351 (100%) | 150 (100%) | 301 (100%) | 210 (100%) | 90 (100%) | 146 (100%) | 49 (100%) | 0 | 171 (100%) | 0 | 0 | 50 (100%) | 241 (100%) | 471 (100%) | 0 |
| **p53 status** |  |  |  |  |  |  |  |  |  |  |  |  |  |  |  |  |
| Mutant | 0 | 0 | 0 | 35 (11,6%) | 0 | 0 | 0 | 0 | 0 | 0 | 0 | 0 | 0 | 0 | 0 | 0 |
| Wild-type | 0 | 0 | 0 | 266 (88,4%) | 0 | 0 | 0 | 0 | 0 | 0 | 0 | 0 | 0 | 0 | 0 | 0 |
| Missing data | 219 (100%) | 351 (100%) | 150 (100%) | 0 | 210 (100%) | 90 (100%) | 146 (100%) | 49 (100%) | 61 (100%) | 171 (100%) | 49 (100%) | 80 (100%) | 50 (100%) | 241 (100%) | 471 (100%) | 95 (100%) |
| **MSI/MSS status** |  |  |  |  |  |  |  |  |  |  |  |  |  |  |  |  |
| MSI | 0 | 59 (16,8%) | 2 (1,3%) | 0 | 0 | 0 | 3 (2,1%) | 0 | 12 (19,7%) | 0 | 7 (14,3%) | 5 (6,3%) | 0 | 0 | 0 | 7 (7,4%) |
| MSS | 0 | 282 (80,3%) | 144 (96%) | 0 | 0 | 0 | 140 (95,9%) | 0 | 49 (80,3%) | 0 | 42 (85,7%) | 75 (93,8%) | 0 | 0 | 0 | 88 (92,6%) |
| Missing data | 219 (100%) | 10 (2,8%) | 4 (2,7%) | 301 (100%) | 210 (100%) | 90 (100%) | 3 (2,1%) | 49 (100%) | 0 | 171 (100%) | 0 | 0 | 50 (100%) | 241 (100%) | 471 (100%) | 0 |
| **Neural invasion** |  |  |  |  |  |  |  |  |  |  |  |  |  |  |  |  |
| Yes | 0 | 61 (17,4%) | 0 | 0 | 0 | 0 | 18 (12,3%) | 0 | 19 (31,1%) | 0 | 7 (14,3%) | 15 (18,8%) | 0 | 0 | 0 | 75 (78,9%) |
| No | 0 | 248 (70,7%) | 0 | 0 | 0 | 0 | 97 (66,4%) | 0 | 42 (68,9%) | 0 | 2 (4,1%) | 7 (8,8%) | 0 | 0 | 0 | 20 (21,1%) |
| Missing data | 219 (100%) | 42 (12%) | 150 (100%) | 301 (100%) | 210 (100%) | 90 (100%) | 31 (21,2%) | 49 (100%) | 0 | 171 (100%) | 40 (81,6%) | 58 (72,5%) | 50 (100%) | 241 (100%) | 471 (100%) | 0 |
| **Vascular invasion** |  |  |  |  |  |  |  |  |  |  |  |  |  |  |  |  |
| Yes | 0 | 104 (29,6%) | 0 | 0 | 0 | 0 | 24 (16,4%) | 0 | 5 (31,1%) | 0 | 19 (38,8%) | 23 (28,8%) | 0 | 0 | 0 | 34 (35,8%) |
| No | 0 | 214 (61%) | 0 | 0 | 0 | 0 | 101 (69,2%) | 0 | 56 (91,8%) | 0 | 3 (6,1%) | 7 (8,8%) | 0 | 0 | 0 | 61 (64,2%) |
| Missing data | 219 (100%) | 33 (9,4%) | 150 (100%) | 301 (100%) | 210 (100%) | 90 (100%) | 21 (14,4%) | 49 (100%) | 0 | 171 (100%) | 27 (55,1%) | 50 (62,5%) | 50 (100%) | 241 (100%) | 471 (100%) | 0 |
| **Smoking history** |  |  |  |  |  |  |  |  |  |  |  |  |  |  |  |  |
| Current smoker | 0 | 0 | 0 | 0 | 74 (35,2%) | 53 (58,9%) | 0 | 0 | 0 | 81 (47,4%) | 0 | 0 | 0 | 0 | 0 | 0 |
| Non-current smoker | 0 | 0 | 0 | 0 | 24 (11,4%) | 37 (41,1%) | 0 | 0 | 0 | 90 (52,6%) | 0 | 0 | 0 | 0 | 0 | 0 |
| Missing data | 219 (100%) | 351 (100%) | 150 (100%) | 301 (100%) | 112 (53,3%) | 0 | 146 (100%) | 49 (100%) | 61 (100%) | 0 | 49 (100%) | 80 (100%) | 50 (100%) | 241 (100%) | 471 (100%) | 95 (100%) |
| **Neoadjuvant treatment** |  |  |  |  |  |  |  |  |  |  |  |  |  |  |  |  |
| Yes | 0 | 1 (0,3%) | 0 | 0 | 7 (3,3%) | 0 | 86 (58,95%) | 0 | 0 | 0 | 0 | 0 | 0 | 0 | 0 | 0 |
| No | 0 | 347 (98,9%) | 0 | 0 | 0 | 90 (100%) | 48 (32,9%) | 0 | 61 (100%) | 171 (100%) | 0 | 0 | 50 (100%) | 0 | 471 (100%) | 95 (100%) |
| Missing data | 219 (100%) | 3 (0,9%) | 150 (100%) | 301 (100%) | 203 (96,7%) | 0 | 12 (8,2%) | 49 (100%) | 0 | 0 | 49 (100%) | 80 (100%) | 0 | 241 (100%) | 0 | 0 |
| **Adjuvant treatment** |  |  |  |  |  |  |  |  |  |  |  |  |  |  |  |  |
| Yes | 0 | 126 (35,9%) | 72 (48%) | 275 (91,4%) | 58 (27,6%) | 40 (44,4%) | 28 (19,2%) | 46 (93,9%) | 18 (29,5%) | 65 (38%) | 3 (6,1%) | 8 (10%) | 43 (86%) | 40 (16,6%) | 416 (88,3%) | 51 (53,7%) |
| No | 0 | 132 (37,6%) | 1 (0,7%) | 26 (8,6%) | 0 | 45 (50%) | 94 (64,4%) | 3 (6,1%) | 43 (70,5%) | 90 (52,6%) | 46 (93,9%) | 72 (90%) | 7 (14%) | 40 (16,6%) | 55 (11,7%) | 44 (46,3%) |
| Missing data | 219 (100%) | 93 (26,5%) | 77 (51,3%) | 0 | 152 (72,4%) | 5 (5,6%) | 24 (16,4%) | 0 | 0 | 16 (9,4%) | 0 | 0 | 0 | 161 (66,8%) | 0 | 0 |

^a^Median survival times were calculated using the Kaplan-Meier method

^b^Mean survival times were estimated when median survival times cannot be calculated

**Supplementary Table 2: Univariable analysis using a Cox proportional hazards regression model with stroma fraction dichotomized through median cut-off.** Hazard ratios (HR) for overall survival are presented with 95% confidence interval (95% CI).

| TUMOR code | HR | 95% CI | p value | n | Tumour type |
| --- | --- | --- | --- | --- | --- |
| RCC | 1.11 | 0.76-1.61 | 0.580 | 219 | Renal Cell Cancer |
| CC | 1.08 | 0.81-1.45 | 0.583 | 351 | Colon Cancer |
| OVC | 1.04 | 0.73-1.50 | 0.596 | 150 | Ovarian Carcinoma |
| ENC | 0.84 | 0.53-1.32 | 0.450 | 301 | Endometrial Cancer |
| UBC | 1.48 | 1.07-2.05 | 0.018 | 210 | Urine Bladder Cancer |
| LUSC | 0.60 | 0.36-0.99 | 0.044 | 90 | Lung Squamous Cell Cancer |
| RC | 0.78 | 0.48-1.28 | 0.325 | 146 | Rectal Cancer |
| HGSC | 0.75 | 0.41-1.38 | 0.355 | 49 | High Grade Serous Ovarian Cancer |
| PACi * | 2.61 | 1.22-5.61 | 0.014 | 61 | Periampullary Cancer, Intestinal type |
| LUAD | 0.88 | 0.61-1.28 | 0.517 | 171 | Lung Adenocarcinoma |
| SC | 1.33 | 0.71-2.5 | 0.377 | 49 | Stomach Cancer |
| GECA | 0.80 | 0.48-1.33 | 0.388 | 80 | Gastroesophageal Junction Adenocarcinoma |
| BRC ER- | 0.62 | 0.29-1.3 | 0.205 | 50 | Breast Cancer, ER-negative |
| PC | 0.70 | 0.42-1.15 | 0.168 | 241 | Prostate Cancer |
| BRC ER+ | 0.85 | 0.63-1.15 | 0.303 | 471 | Breast Cancer, ER-positive |
| PACpb | 0.84 | 0.55-1.31 | 0.448 | 95 | Periampullary Cancer, Pancreatobiliary type |

* Due to violation of proportional hazards assumption a modified model split by time segments (0 to 70, and 70 to max) was applied for **PACi:**

| Time segment | HR | 95% CI | p value |
| --- | --- | --- | --- |
| <70 weeks | 1.10 | 0.32-3.81 | 0.878 |
| >70 weeks | 4.28 | 1.55-11.88 | 0.005 |

**Supplementary Table 3:** **Univariable analysis using a Cox proportional hazards regression model with stroma fraction used as continuous value, rounded to first decimal.** Hazard ratios (HR) for overall survival are presented with 95% confidence interval (95% CI).

| TUMOR code | HR | 95% CI | p value | n | Tumour type |
| --- | --- | --- | --- | --- | --- |
| RCC | 1.18 | 0.56-2.52 | 0.661 | 219 | Renal Cell Cancer |
| CC | 0.88 | 0.42-1.85 | 0.735 | 349 | Colon Cancer |
| OVC | 1.35 | 0.91-1.99 | 0.130 | 150 | Ovarian Carcinoma |
| ENC | 0.92 | 0.26-3.19 | 0.890 | 301 | Endometrial Cancer |
| UBC | 3.15 | 1.60-6.19 | 0.001 | 210 | Urine Bladder Cancer |
| LUSC | 0.21 | 0.06-0.77 | 0.018 | 90 | Lung Squamous Cell Cancer |
| RC | 0.46 | 0.14-1.53 | 0.206 | 146 | Rectal Cancer |
| HGSC | 0.74 | 0.18-3.09 | 0.676 | 49 | High Grade Serous Ovarian Cancer |
| PACi * | 20.2 | 3.25-125. | 0.001 | 61 | Periampullary Cancer, Intestinal type |
| LUAD | 0.61 | 0.23-1.57 | 0.303 | 171 | Lung Adenocarcinoma |
| SC | 1.52 | 0.36-6.45 | 0.568 | 49 | Stomach Cancer |
| GECA | 0.35 | 0.09-1.31 | 0.119 | 80 | Gastroesophageal Junction Adenocarcinoma |
| BRC ER- | 0.2 | 0.03-1.46 | 0.111 | 50 | Breast Cancer, ER-negative |
| PC | 0.24 | 0.05-1.22 | 0.086 | 235 | Prostate Cancer |
| BRC ER+ | 0.48 | 0.22-1.05 | 0.067 | 470 | Breast Cancer, ER-positive |
| PACpb | 0.53 | 0.12-2.43 | 0.417 | 95 | Periampullary Cancer, Pancreatobiliary type |

* Due to violation of proportional hazards assumption a modified model split by time segments (0 to 70, and 70 to max) was applied for **PACi:**

| Time segment | HR | 95% CI | p value |
| --- | --- | --- | --- |
| <70 weeks | 6.27 | 0.27-144.54 | 0.251 |
| >70 weeks | 36.8 | 3.83-354.35 | 0.002 |

**References**

1. Glimelius B, Melin B, Enblad G, Alafuzoff I, Beskow A, Ahlstrom H, et al. U-CAN: a prospective longitudinal collection of biomaterials and clinical information from adult cancer patients in Sweden. Acta Oncol. 2018;57(2):187-94.

2. Elebro J, Jirstrom K. Use of a standardized diagnostic approach improves the prognostic information of histopathologic factors in pancreatic and periampullary adenocarcinoma. Diagnostic pathology. 2014;9:80.

3. Elebro J, Heby M, Warfvinge CF, Nodin B, Eberhard J, Jirstrom K. Expression and Prognostic Significance of Human Epidermal Growth Factor Receptors 1, 2 and 3 in Periampullary Adenocarcinoma. PloS one. 2016;11(4):e0153533.

4. Berglund G, Elmstahl S, Janzon L, Larsson SA. The Malmo Diet and Cancer Study. Design and feasibility. J Intern Med. 1993;233(1):45-51.

5. Nodin B, Zendehrokh N, Brandstedt J, Nilsson E, Manjer J, Brennan DJ, et al. Increased androgen receptor expression in serous carcinoma of the ovary is associated with an improved survival. J Ovarian Res. 2010;3:14.

6. Berglund G, Eriksson KF, Israelsson B, Kjellstrom T, Lindgarde F, Mattiasson I, et al. Cardiovascular risk groups and mortality in an urban swedish male population: the Malmo Preventive Project. J Intern Med. 1996;239(6):489-97.

7. Moyano-Galceran L, Pietila EA, Turunen SP, Corvigno S, Hjerpe E, Bulanova D, et al. Adaptive RSK-EphA2-GPRC5A signaling switch triggers chemotherapy resistance in ovarian cancer. EMBO Mol Med. 2020;12(4):e11177.

8. Frodin M, Mezheyeuski A, Corvigno S, Harmenberg U, Sandstrom P, Egevad L, et al. Perivascular PDGFR-beta is an independent marker for prognosis in renal cell carcinoma. Br J Cancer. 2017;116(2):195-201.

9. Sjoberg E, Frodin M, Lovrot J, Mezheyeuski A, Johansson M, Harmenberg U, et al. A minority-group of renal cell cancer patients with high infiltration of CD20+B-cells is associated with poor prognosis. Br J Cancer. 2018;119(7):840-6.

10. Hemdan T, Linden M, Lind SB, Namuduri AV, Sjostedt E, de Stahl TD, et al. The prognostic value and therapeutic target role of stathmin-1 in urinary bladder cancer. Br J Cancer. 2014;111(6):1180-7.

11. Edqvist PH, Huvila J, Forsstrom B, Talve L, Carpen O, Salvesen HB, et al. Loss of ASRGL1 expression is an independent biomarker for disease-specific survival in endometrioid endometrial carcinoma. Gynecol Oncol. 2015;137(3):529-37.

12. Huvila J, Laajala TD, Edqvist PH, Mardinoglu A, Talve L, Ponten F, et al. Combined ASRGL1 and p53 immunohistochemistry as an independent predictor of survival in endometrioid endometrial carcinoma. Gynecol Oncol. 2018;149(1):173-80.

13. Micke P, Mattsson JS, Djureinovic D, Nodin B, Jirstrom K, Tran L, et al. The Impact of the Fourth Edition of the WHO Classification of Lung Tumours on Histological Classification of Resected Pulmonary NSCCs. J Thorac Oncol. 2016;11(6):862-72.

14. Svensson MC, Warfvinge CF, Fristedt R, Hedner C, Borg D, Eberhard J, et al. The integrative clinical impact of tumor-infiltrating T lymphocytes and NK cells in relation to B lymphocyte and plasma cell density in esophageal and gastric adenocarcinoma. Oncotarget. 2017;8(42):72108-26.

15. Svensson MC, Borg D, Zhang C, Hedner C, Nodin B, Uhlen M, et al. Expression of PD-L1 and PD-1 in Chemoradiotherapy-Naive Esophageal and Gastric Adenocarcinoma: Relationship With Mismatch Repair Status and Survival. Front Oncol. 2019;9:136.

16. Krzyzanowska A, Don-Doncow N, Marginean FE, Gaber A, Watson RW, Hellsten R, et al. Expression of tSTAT3, pSTAT3(727) , and pSTAT3 (705) in the epithelial cells of hormone-naive prostate cancer. Prostate. 2019;79(7):784-97.
